# Hyperlipidemias in elderly patients: results from the Berlin Aging Study II (BASEII), a cross-sectional study

**DOI:** 10.1186/s12944-020-01277-9

**Published:** 2020-05-14

**Authors:** Adrian Rosada, Ursula Kassner, Felix Weidemann, Maximilian König, Nikolaus Buchmann, Elisabeth Steinhagen-Thiessen, Dominik Spira

**Affiliations:** 1 Department of Geriatrics, Charité Universitätsmedizin Berlin, Berlin, Germany; 2 Department of Endocrinology and Metabolic Diseases (including Lipid Metabolism), Charité Universitätsmedizin Berlin, Berlin, Germany; 3 Faculty of Mathematics, Informatics and Statistics, Ludwig-Maximilians-University, Munich, Germany; 4 Department of Cardiology, Charité-Universitätsmedizin Berlin, Berlin, Germany

**Keywords:** Hyperlipidemia, prevalence, treatment targets, elderly patients, cardiovascular disease, Lipoprotein(a)

## Abstract

**Background:**

Hyperlipidemias are common and the last decades have seen substantially growing evidence of their causative role in the development of atherosclerosis and subsequent cardiovascular diseases. Since hyperlipidemias usually do not cause direct clinical symptoms, they often remain undiagnosed until a serious cardiovascular event occurs. Especially for LDL-hypercholesteremia, there are well-established treatment options available to prevent the occurrence of atherosclerosis. However, there is a lack of knowledge regarding the proper treatment of elderly patients. The goal of this study was to assess the prevalence of hyperlipidemia in a group of young and a group of elderly community-dwelling participants and to determine to what extent treatment of hyperlipidemia should be initiated or required.

**Methods:**

Crossectional data from a total of 2151 subjects (1657 in the elderly group, mean age 69, and 494 in the young group (control group), mean age 29) of the Berlin Aging Study II (BASE-II) were available. Medical history was assessed and recorded by trained physicians and prevalence of lipid disorders was determined with laboratory tests, including a lipid-profile.

**Results:**

A large proportion of subjects (39%) were unaware of an existing lipid disorder. The prevalence of hyperlipidemia was more frequent in the elderly group (76%) compared to the young group (41%). Hypercholesterolemia was the most common diagnosed disorder (64%), followed by hyperlipoproteinemia(a) (18%), hypertriglyceridemia (7%) and combined hyperlipoproteinaemia (5%). Only a minority of this cohort was treated with lipid-lowering medication (17%) and of those treatment targets according to ESC guidelines were reached only in 16.5 %.

**Conclusions:**

Hyperlipidemias appear underdiagnosed and undertreated. As the prevalence of these disorders increases with age and with regard to their role as a major modifiable risk factor for cardiovascular disease it seems to be advisable to aim for more consistent and sustainable screening and treatment of these common disorders.

**Trial Registration:**

BASE-II registered with the clinical trial registry Deutsches Register Klinischer Studien (DRKS00009277).

## Introduction

Hyperlipidemias are common and particularly LDL-Hypercholesterolemia plays a causative role in the pathogenesis of cardiovascular diseases such as coronary heart disease and cerebro-vascular disease [1, 2]. The most common hyperlipidemia are hypercholesterolemia, hypertriglyceridemia, combined hyperlipoproteinaemia, and hyperlipoproteinemia(a). Of those, hypercholesterolemia based on elevated LDL-cholesterol-levels is the best analyzed and understood disease with the most effective treatment options. Less data are available on the role of hypertriglyceridemia, combined hyperlipoproteinaemia and hyperlipoproteinemia(a) in the pathogenesis of cardiovascular disease, therefore most recommendations are less specific in these disorders and at the same time treatment options are limited. It is generally accepted that treatment of high cholesterol levels should be performed based on risk stratification with assessment of other co-existing risk factors including age, sex, smoking, hypertension, diabetes or chronic kidney disease [3, 4]. Moreover, general consensus exists for treatment with statins when coronary heart disease, stroke and peripheral arterial disease already present to prevent atherosclerotic plaque progression and recurrent events. Although it is unclear which treatment target levels should specifically be achieved in old and very old patients (>80 years), it appears that patients in general benefit rather from moderate to high-intensive statin therapy compared to low-intensive statin therapy [4]. With regard to primary prevention (patients who do not suffer from coronary heart disease, stroke and peripheral arterial disease) it is recommended to treat patients in certain risk groups such as patients with very high levels of LDL-cholesterol (>190 mg/dL), coexisting diabetes mellitus or with an intermediate or high 10-year risk to suffer major cardiovascular events [5, 6].

The goal of this study was to assess the prevalence of hyperlipidemia in a group of young (mean age 29 years) and a group of elderly community-dwelling participants (mean age 68 years) and to determine to what extent treatment of hyperlipidemia should be initiated or required.

## Methods

Subjects were participants of the Berlin Aging Study II (BASE-II), which comprises about 2.151 adult volunteers from the Berlin metropolitan area [7]. The BASE-II study and the participant recruitment have been described in detail in previous publications [7, 8]. BASE-II is a prospective epidemiological study aimed at the identification and characterization of factors associated with “healthy” *vs* “unhealthy” ageing, broadly examining individuals in domains such as mental and physical health, psychological functioning and social as well as economic status. In short, eligibility criteria at the time of recruitment were community-dwelling elderly subjects aged between 60 and 82 years for the elderly group. All participants were examined by trained physicians who assessed the medical history and the current and previous medication. In addition to recording disease states, functional status was assessed with validated questionnaires and a comprehensive geriatric assessment. Comparisons with representative survey data from Berlin and Germany revealed that BASE-II participants are characterized by slightly higher education and better self-reported health status than the general population of Berlin and Germany [7].

### Anthropometric measurements:

Body weight was measured in light clothes with a portable electronic scale to the nearest 0.1 kg and height was determined to the nearest 0.1 cm by using an electronic weighing and measuring station (seca 764, seca, Hamburg, Germany). Weight and height were used to calculate the body mass index (BMI) (weight [kg]/height [m]^2^).

### Functional tests:

Handgrip strength was assessed with a Smedley Dynamometer (Scandidact, Denmark). The subjects were instructed to perform a maximal isometric contraction, the test was performed three times for each hand and the highest value of either side was chosen.

### Laboratory testing:

Total cholesterol, LDL-cholesterol, HDL-cholesterol and triglycerides were measured via a homogeneous enzymatic colorimetric assay (Cobas®; Manufacturer: Roche Diagnostics GmbH, Sandhafer Strasse 116, 68305 Mannheim; Germany)

Lipoprotein(a) was measured via a particle enhanced immunoturbidimetric test. It uses a fixed time determination of the Lp(a) concentration by photometric measurement of antigen-antibody-reaction between antibodies against Lp(a) bound to particles and Lp(a) present in the sample. (Cobas®; Manufacturer: Roche Diagnostics GmbH, Sandhafer Strasse 116, 68305 Mannheim; Germany)

### Criteria for lipid-disorders were (fasting measurement):

Hypercholesterolemia: total Cholesterol >= 5.2 mmol/L (200 mg/dL) [9]

Combined hyperlipoproteinemia: total Cholesterol >= 5.2 mmol/L and triglycerides >= 2.28 mmol/L (200 mg/dL) [10]

Low HDL Cholesterol: HDL Cholesterol <1.0 mmol/L in men or <1.3 mmol/L in women [6]

Hypertriglyceridemia: Triglycerides >= 2.28 mmol/L [10]

Hyperlipoproteinemia(a): Lipoprotein(a) >= 50 mg/dL [11].

### Statistical analysis

In the current study cross-sectional data were used to determine the prevalence of certain diseases in the two BASE-II age groups. In addition, medians of laboratory findings were compared. Data are presented as median and inter quartile range (IQR). As laboratory findings were not normally distributed Kolmogorov–Smirnov test was used to compare differences between groups. To compare prevalence/proportions the chi-square test was used. Statistical analyses were conducted using IBM® SPSS® Statistics Version 23. A critical alpha level (i.e., *P*-value) of <0.05 was employed to indicate statistical significance.

## Results

### Demographic data

The mean age of subjects was 29.0 ± 3.2 and 68.8 ± 3.7 years in the young age and the elderly group respectively. The sex distribution was comparable in both groups (53% female subjects in the young group, 51% female subjects in the elderly group).

The mean body mass index was 23.3 kg/m^2^ in the young group and 26.9 kg/m^2^ in the elderly group, while the mean waist circumference was 82 cm in the young group and 96 cm in the elderly group (see Table 1). In the elderly group 18% reported to have consulted an internist during the last three months, 60% had consulted a general practitioner. In the young group 5% reported to have consulted an internist during the last three months, 35% had consulted a general practitioner.

**Table 1:** Demographic data, prevalence of diseases, health assessment in both groups

Study Group	Young Age Group	Elderly Age Group
Total Number (n) or mean (SD)	494	1657
Age years mean (SD (standard deviation))	29.0 (3.2)	68.8 (3.7)
Female	53.0%	51.4%
Arterial Hypertension (systolic BP >=140 mmHg and/or diastolic BP >=90 mmHg)	14.6%	72.7%
Diabetes	0.2%	11.5%
Current smoker	31.2%	9.3%
Body mass index (SD) kg/m^2^	23.3 (4.1)	26.9 (4.2)
Waist circumference cm (SD)	82 (12)	96 (12)
Chronic kidney disease (GFR <= 60mL/min/1.73m^2^)	0.0%	17.1%
Coronary heart disease or equivalent	0.0%	7.5%
Global Self-Assessment of Health Status “good” or “very good”	85.4%	71.6%
Self-Assessment of Health Status from the 36-Item Short Form Survey (SF-36) “good” or better	-	85.5%
Global Self-Assessment of Satisfaction with Life (0= completely dissatisfied to 10= completely satisfied) (SD)	7.6 (1.3)	7.9 (1.5)
Right Hand Grip Strength (SD) kg	38.2 (10.1)	33.6 (9.7)

Abbreviations: BP: blood pressure, GFR: glomerular filtration rate, SD: standard deviation, SF-36: Self-Assessment of Health Status from the 36-Item Short Form Survey

### Prevalence of diseases

Chronic diseases were more common in the elderly group: Arterial hypertension was present in 73% of the elderly group and in 15% of the young group. Diabetes was diagnosed in 12% of subjects from the elderly group and 0% in the young group. Chronic kidney disease (GFR (glomerular filtration rate) <= 60mL/min/1.73m^2^ ) was present in 17% of the elderly group and 0% in the young group, while a coronary heart disease or equivalent (history of myocardial infarction, peripheral artery disease or cerebrovascular disease) was known in 8% of the elderly group and 0% in the young group. 31% of the young group were current smokers and 19% former smokers, while 9% of the elderly group were current smokers and 43% former smokers.

### Health Assessment

Elderly subjects showed comparable results to young subjects in regard of self-assessment of general health, while maximal handgrip strength was lower (34 *vs*. 38 kg elderly *vs*. young group).

### Hyperlipidemia

In regard of hyperlipidemia, only 3% of the young group reported to have a known lipid disorder, while 37% of the elderly group reported to have a lipid disorder.

The elderly group showed significantly higher levels of total cholesterol, LDL cholesterol, triglycerides and lipoprotein(a) compared to the young group (see Table 2). Transferred to diagnoses the elderly group met significantly more often the criteria for hypercholesterolemia, hypertriglyceridemia, combined hyperlipoproteinemia, and hyperlipoproteinemia(a) compared to the young group. There were no significant differences between groups in regard of HDL-cholesterol (11.0% *vs*. 13.0%), although there was the known significant difference between female and male subjects (females having higher levels compared to males) seen similar in all groups (young *vs*. elderly; untreated *vs*. treated).

**Table 2:** Laboratory findings, prevalence of lipid disorder and treatments in both groups

Study Group	Young Age Group		Elderly Age Group		***P***
Total Number (n)	494		1657		
Reported Lipid Disorder	2.6%		37.1%		<0.01
**Laboratory findings**					
Total Cholesterol, median (IQR (interquartile range)), mmol/L	4.5 (4.0 – 5.1)		5.6 (4.9 – 6.3)		<0.01
LDL-Cholesterol, median (IQR), mmol/L	2.5 (2.0 – 3.0)		3.4 (2.8 – 4.0)		<0.01
HDL-Cholesterol, median (IQR),all, mmol/L	1.5 (1.3 – 1.8)		1.6 (1.3 – 1.9)		0.097
HDL-Cholesterol, median (IQR), by sex, mmol/L	female 1.8 (1.5–2.0)	male 1.4 (1.1-1.6)	female 1.8 (1.5-2.0)	male 1.4 (1.1-1.7)	<0.01/<0.01
Lipoprotein(a), median (IQR), mg/dL	7.0 (1.5 – 21.0)		11.0 (4.6 – 33.9)		<0.01
Triglycerides, median (IQR), mmol/L	0.9 (0.7 – 1.2)		1.1 (0.9 – 1.5)		<0.01
**Any measured Lipid Disorder**	40.6%		76.0%		<0.01
- Hypercholesterolemia (Total Cholesterol >= 5.2 mmol/L)	23.7%		64.0%		<0.01
- Hypertriglyceridemia (Triglycerides >= 2.3 mmol/L)	3.5%		6.8%		<0.01
- Combined Hyperlipoproteinaemia (Total Cholesterol >= 5.2 mmol/L and Triglycerides >= 2.3 mmol/L)	2.6%		4.6%		0.054
- Hyperlipoproteinemia(a) (lipoprotein(a) >= 50 mg/dL)	7.9%		18.3%		<0.01
- Low HDL Cholesterol (HDL Cholesterol <1.0 mmol/L in men or <1.3 mmol/L in women), all	13.0%		11.0%		0.238
- Low HDL Cholesterol (HDL Cholesterol <1.0 mmol/L in men or <1.3 mmol/L in women), by sex	female 11.6%	male 14.5%	female 9.8%	male 12.3%	0.342/0.107
- Combined Hypertriglyceridemia and Low HDL Cholesterol	2.3%		3.1%		0.356
**Consultation of a general practitioner during the last 3 months**	35.0%		60.3%		<0.01
**Treated with any lipid-lowering medication**	0.0%		16.8%		<0.01
- Statins	0.0%		16.3%		<0.01
- Fibrates	0.0%		0.2%		0.274
- Ezetimibe	0.0%		1.1%		0.02
**LDL-treated to target, ESC-Guidelines**	97.6%		7.4%		<0.01

Abbreviations: ESC: European Society of Cardiology, GFR: glomerular filtration rate, HDL: high density lipoprotein, IQR: interquartile range, LDL: low density lipoprotein, Lp(a): Lipoprotein(a), SD: standard deviation

### Combined lipid disorders

In both groups elevated triglyzerides were found in about two thirds combined with elevated LDL-cholesterol, showing that elevated triglycerides commonly appear in form of a combined hyperlipoproteinemia. 5% of subjects with hyperlipoproteinemia(a) had co-existing hypertriglyceridemia in both age groups. In regard of the coexistence of hypercholesterolemia and hyperlipoproteinemia(a) there was a difference between the age groups: while 73.0% of elderly subjects with a hyperlipoproteinemia(a) had a co-existing hypercholesterolemia, only 43.1% of young subjects with a hyperlipoproteinemia(a) had co-existing hypercholesterolemia.

The combination of hypertriglyceridemia and Low HDL cholesterol was present in 2.3% in the young group and 3.1% in the elderly group.

### Findings in relation to current guidelines

**Fig. 1:**
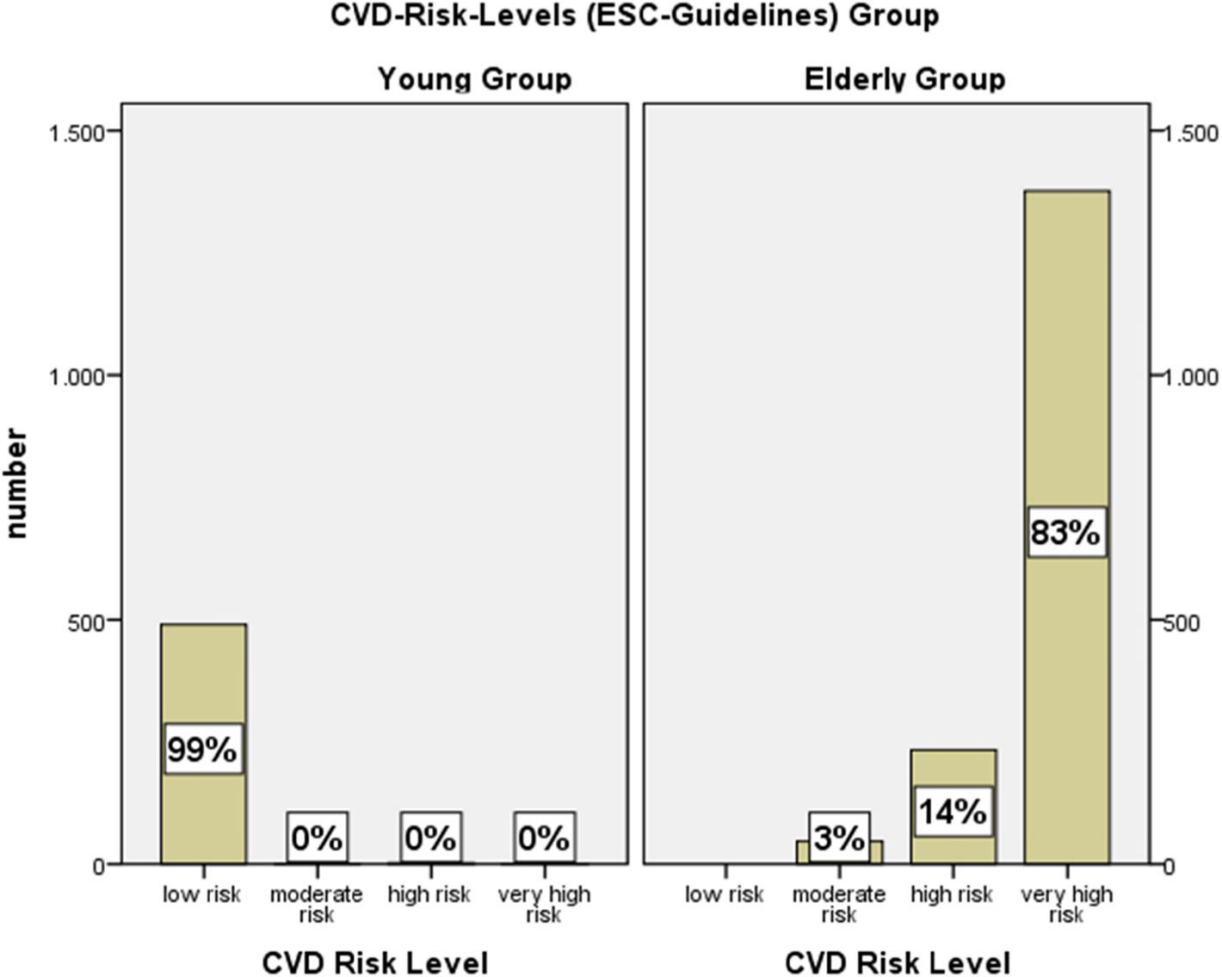
Frequency of the 4 risk levels among subjects in both groups to suffer a fatal CVD (cardio vascular disease) in the next 10 years, defined in the ESC/EAS (European Society of Cardiology/ European Atherosclerosis Society) Guidelines for the management of dyslipidaemia

The European guidelines on prevention of cardiovascular disease in clinical practice (2016) define 4 different risk groups for the risk to suffer a fatal cardiovascular disease in the next 10 years (low risk, moderate risk, high risk, very high risk) [2, 3]. The calculation of these risk groups uses a formula which includes sex, age, measured blood pressure, measured cholesterol levels and smoking status. It is based on large cohort studies with cardiovascular events. The result of the formula is the total 10-year risk of a fatal cardiovascular disease given in percent [12]. 84% of the elderly group belonged to the very high risk group to suffer a fatal cardiovascular disease in the next 10 years, 13% belonged to the high risk group, and 3% to the moderate risk group (see Figure 1). In the young group 99% belonged to the low risk group and 1% to the high-risk group (mainly due to obesity, hypertension, diabetes, and smoking).

**Fig. 2:**
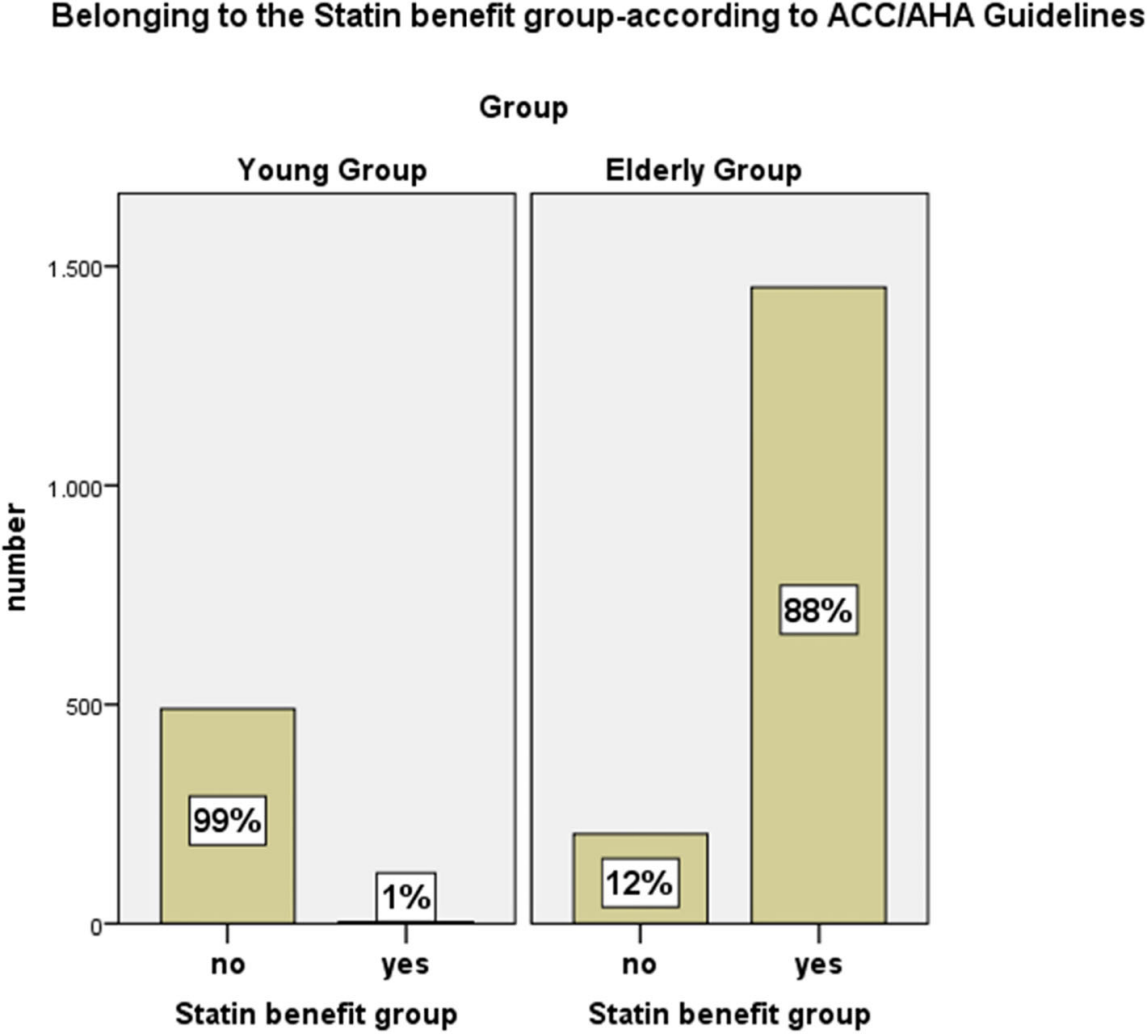
Frequency of belonging to the “Statin benefit group”, defined in the ACC/AHA Guidelines (American College of Cardiology/American Heart Association) on the Treatment of Blood Cholesterol to Reduce Atherosclerotic Cardiovascular Risk in Adults compared in both groups

The American College of Cardiology/American Heart Association Guidelines on the Treatment of Blood Cholesterol to Reduce Atherosclerotic Cardiovascular Risk takes a slightly different approach and defines “Statin benefit groups” considering prevalent diseases including diabetes, myocardial infarction or equivalent, and levels of LDL-cholesterol [4]. The relevant conditions assigning patients to “Statin benefit groups” are: 1. Prevalent atherosclerotic cardiovascular disease or 2. Severe hypercholesterolemia (LDL-C ≥4.9 mmol/L) or 3. Diabetes mellitus or 4. An intermediate or higher risk (≥7.5%) for atherosclerotic cardiovascular disease in patients in primary prevention. According to this 88% of the elderly group belong to a statin benefit group, while 1% of the young group belong to the statin benefit group (see Figure 2).

Lipid treatment and goals While none in the young group was treated with lipid lowering medication, 17% of the elderly group were on treatment. This treatment consisted mainly of statins (16%) followed by ezetimibe (1%) and fibrates (0.2%). Regarding the LDL treatment targets, only 7% of the elderly group were treated to target while 98% of the young group had LDL-levels at target.

Subjects of the elderly group were divided in subjects treated with lipid-lowering medication and subjects without lipid lowering medication. Subjects on lipid-lowering medication showed significantly lower levels of total cholesterol, LDL-cholesterol and HDL-cholesterol compared to subjects without treatment. At the same time subjects on lipid-lowering medication showed significantly higher levels of triglycerides and lipoprotein(a). Subjects receiving lipid-lowering medication showed higher prevalence of arterial hypertension, diabetes, chronic kidney disease and coronary heart disease or equivalent. At the same time participants receiving lipid-lowering medication were more likely male than female (58.4% *vs*. 46.6% in the non-treatment group). 17% of the patients on treatment reached LDL-goals, while 6% of the untreated patients had LDL-cholesterol levels at goal (see Table 3).

**Table 3:** Laboratory findings in treated and untreated subjects of the elderly group

Study Group	Not treated with any lipid-lowering medication		Treated with any lipid-lowering medication		***P***
Total Number (n)/Percentage (%)	1357 (83.2%)		273 (16.8%)		
Male	46.6%		58.4 %		<0.01
Current smoker	9.0%		10.8%		0.338
Consultation of a general practitioner during the last 3 months	58.3%		70.5%		<0.01
Arterial Hypertension (systolic BP >=140 mmHg and/or diastolic BP >=90 mmHg)	70.5%		83.2%		<0.01
Diabetes mellitus	9.1%		22.9%		<0.01
Chronic kidney disease (GFR <= 60mL/min/1,73m^2^)	6.7%		12.8%		<0.01
Coronary heart disease or equivalent	4.2%		24.0%		<0.01
**Laboratory findings**					
Total cholesterol, median (IQR), mmol/L	5.7 (5.0 – 6.3)		4.8 (4.2 – 5.4)		<0.01
LDL-Cholesterol, median (IQR), mmol/L	3.5 (2.9 – 4.1)		2.6 (2.1 – 3.2)		<0.01
HDL-Cholesterol, median (IQR), all, mmol/L	1.6 (1.3 – 1.9)		1.5 (1.2 – 1.8)		<0.01
HDL-Cholesterol, median (IQR), by sex, mmol/L	female 1.8 (1.5–2.1)	male1.4 (1.1-1.7)	female 1.7 (1.4-2.0)	male 1.4 (1.1-1.6)	0.04/ <0.16
Lipoprotein(a), median (IQR), mg/dL	11.0 (4.2 – 31.9)		11.0 (5.0 – 41.5)		0.163
Triglycerides, median (IQR), mmol/L	1.1 (0.8 – 1.5)		1.2 (0.9 – 1.7)		<0.01
**LDL-C at target, ESC-Guidelines**	5.6%		16.5%		<0.01

Abbreviations: BP: blood pressure, ESC: European Society of Cardiology, GFR: glomerular filtration rate, HDL: high density lipoprotein, IQR: interquartile range, LDL: low density lipoprotein, Lp(a): Lipoprotein(a), SD: standard deviation

## Discussion

This study highlights that the prevalence of hyperlipidemia increases with age. Significant differences in levels of lipid parameters and prevalence of hyperlipidemia have been observed between the young and the elderly group. The elderly group showed strikingly higher levels of total cholesterol (mean difference 1.1 mmol/l) and fulfilled far more often the diagnostic criteria of hypercholesterolemia (64.0 *vs*. 23.7% old *vs*. young). There are various proposed reasons accounting for these differences, i.e. higher lipid levels in older age, including the age-associated loss of hepatic LDL receptors, higher body-mass index, larger waist circumference and lower sex hormone levels, among others [13–17]. For triglycerides an association with diabetes and central obesity is known, so hyperlipidemia in conjunction with these health conditions have to be considered [18]. No significant differences were found between the serum levels of HDL-cholesterol in elderly and young subjects. As to this, previous studies have been inconsistent: some postulated a negative correlation between age and HDL-cholesterol levels, others found sex-differences in the change of HDL-levels with age [19, 20]. Drugs such as Fibrates or Niacin can raise HDL-levels, however an effect on cardiovascular endpoints or mortality wasn’t shown by studies to date. To current knowledge increasing HDL levels via pharmacological manipulation beyond optimal lipid lowering therapy for secondary prevention is not beneficial [21]. Likely other factors including smoking status, body-mass index and physical activity are more relevant factors in regard of HDL-cholesterol levels than age [22]. Lipoprotein(a) levels were significantly higher in the elderly group compared to the young group, although the majority in the elderly group (82%) had lipoprotein(a)-levels below the cut-off of 50 mg/d, above which Lp(a) is considered as an additional factor that indicates a very high cardiovascular risk. Intra-individually lipoprotein(a)-levels are relatively constant across the lifespan but a correlation between age, low sex hormone levels and high lipoprotein(a)-levels has also been postulated before [23, 24]. In line with this the findings of this study support the hypothesis of an influence of sex hormone levels on lipoprotein(a)-levels, especially low estrogen levels correlate with high LDL-cholesterol and Lipoprotein(a)-levels.

As expected, it has been observed that subjects treated with lipid-lowering medication showed significantly lower levels of total cholesterol, LDL-cholesterol and HDL-cholesterol than those without treatment; at the same time treated subjects showed higher levels of triglycerides. Statins are the most commonly used lipid-lowering drugs. Statins do not have a relevant influence on triglycerides or lipoprotein(a). Presumably subjects who receive a lipid-lowering treatment have more often a combined hyperlipoproteinemia or an additional hyperlipoproteinemia(a) than those who do not receive any medication. Moreover it is known that high triglyceride levels may mimic high total cholesterol levels, depending on the laboratory method used. Therefore, it can be assumed that these patients are being treated more often compared to others. Applying the recommendations of current guidelines for dyslipidemia, 88-97% of this elderly group should be considered for a drug treatment (primarily statins) [3, 4]. The treatment with statins is considered to be effective in primary and secondary prevention, reducing the relative risk in cardio-vascular events in total by 20% [25–28]. Although the guidelines call for a risk–benefit discussion between the patient and physician before the initiation of statin therapy, it remains unclear how this influences the general therapeutic decision. Taking into account the rise of cholesterol levels with age and the demographic change a rising number of patients who, according to current guidelines, meet the criteria for lipid lowering treatment has to be expected [29, 30]. As elderly subjects show 20% higher cholesterol levels in mean compared to young ones, a different cutoff value could be discussed. At the same time, meeting the diagnostic criteria for hypercholesteremia is not equivalent for an urgent treatment. The results show that (elderly) subjects are often unaware of an existing dyslipidemia, as only 37% in the elderly group reported dyslipidemia compared to 75% with a measured dyslipidemia. This lack of awareness for dyslipidemia has also been seen in previous studies [31]. The treatment gap is even larger as only 17% were on active drug treatment, whereas 20% did not receive active drug treatment though having known dyslipidemia. The undertreatment of elderly patients with dyslipidemia has been described in other studies [32–37]. The difference in frequencies of consulting a general practitioner could be a reason (58.3% *vs*. 70.5%; untreated and treated) had contact to a general practitioner in the last 3 month), although this does not explain the whole difference.

If central obesity or diabetes are present there should be an attempt to improve these conditions primarily because this could result in improved lipid levels. However if this fails, and the conditions are on a stable, not improvable state, initiation of lipid-lowering treatment should not be missed according to guidelines. Although it is not known why these participants are not on any active drug treatment, a lack of information can be hypothesized. Further it can be expected that a majority was never exposed to a statin treatment, so that side-effects of a statin therapy may only have a subordinate role. Including the fact that risk factors as arterial hypertension, diabetes, chronic kidney disease, and coronary heart disease are more prevalent in the elderly patient group, it has to be considered that these patients are at higher cardiovascular risk. Overall, the therapeutical gap seems to be increasing with age.

However, the argument of a dispensable treatment in context of polypharmacy and age remains a controversial one. Regarding time to benefit statins seem to show positive effects after 1-2 years of treatment, whereas at the same time statins belong to intensively investigated drugs, repeatedly proving effects on all-cause mortality and major vascular events in secondary prevention [25, 38, 39]. In primary prevention the evidence is less clear as to elderly patients: it seems that elderly patients without previous vascular diseases may not profit from a short treatment of 2-3 years [40–42]. At the same time in most cited studies about 50% of included elderly patients had a history of vascular diseases, so that the proportion of “real” primary prevention patients might be significantly smaller compared to the young patient group.

Waiving a lipid-treatment should be considered comparably to cases in which physicians waive a treatment of arterial hypertension: multimorbid patients with a short life expectancy who will most likely not reach the point of benefit from a strict treatment to target strategy, are more probe to side-effects and who already suffer from serious functional limitations [43]. But with regard to general health assessment and surrogates such as handgrip strength this data do not suggest that a majority of the elderly participants in this study are afflicted by a generally impaired functional health status that could support the lack of active drug treatment.

Our results are subject to limitations. Due to the cross-sectional data structure statements about the differences between the young and the elderly group have to be handled with care.

It is unknown how differences between generations (like attitude towards healthy nutrition, regular exercise) affect lipid levels. Some of the differences might be attributed to a less healthy attitude of the elderly generation and are not to be expected in this degree in the young group in future. In addition, the effect of a lipid lowering therapy in regard to the time and extend atherosclerosis developed could vary individually. Although some studies, show effects after two years of treatment in elderly patients and theses patients are at highest risk of developing a cardiovascular event. The initiation of a lipid-lowering treatment could be too late to have a significant effect in some patients. Since this study shows clearly that the prevalence of dyslipidemias is generally high and also considerably increased within aged persons compared to younger persons, physicians should screen aged persons for hyperlipidemia. Hypercholesterolemia is usually a well controllable risk factor in regard of end-organ damage associated with above-mentioned diseases. Similar to arterial hypertension, which is a disease with an increased prevalence with age, physicians should screen for hyperlipidemia and treat patients based on clinical judgement and an individually patient-centered approach as the treatment can reduce the occurrence of cardiovascular events even in elderly patients. Of course, geriatric aspects such as a potentially higher rate of side effects, especially myalgia and myopathy with potentially can cause also functional limitation and drug interactions in case of polypharmacy must be regarded -In case of statin intolerance ezetimibe or PCSK9 (proprotein convertase subtilisin/kexin type 9 ) inhibitors represent reasonable alternative treatment options. In the end it remains to the patients decision after an information conversation with their treating doctor if they accept to be on active treatment because of high lipid levels, comparable to be on treatment because of hypertension.

## Conclusion

### Clinical relevance

Hyperlipidemia is more prevalent in elderly patients compared to young patients and appear to be undertreated. As hyperlipidemia can be diagnosed and treated effectively, and since treatment with statins reduces the occurrence of cardiovascular events in total by up to 20%, treatment should not be withhold from patients solely due to their age alone.

### Future perspective

More information is needed in elderly patients to adjust treatment options, and to select those with the highest potential benefit of drug therapy.

### Take home message

Physicians should screen elderly patients regularly for hyperlipidemia and hyperlipidemia should be treated according to guidelines and good clinical practice.

## Data Availability

Due to concerns for participant privacy, data are available only upon request. External scientists may apply to the Steering Committee of BASE-II for data access. Please contact Dr. Ludmila Müller at lmueller@mpib-berlin.mpg.de

## Abbreviations

ACC: American College of Cardiology
AHA: American Heart Association
BASE-II: Berlin Aging Study II
BMI: Body mass index
CVD: Cardio vascular disease
DRKS: Deutsches Register Klinischer Studien
EAS: European Atherosclerosis Society
ESC: European Society of Cardiology
GFR: Glomerular filtration rate
HDL: High density lipoprotein
IQR: Interquartile range
LDL: Low density lipoprotein
Lp(a): Lipoprotein(a)
PCSK9: Proprotein convertase subtilisin/kexin type 9
SD: Standard deviation
SF-36: Self-Assessment of Health Status from the 36-Item Short Form Survey
